# SESTRINs: Emerging Dynamic Stress-Sensors in Metabolic and Environmental Health

**DOI:** 10.3389/fcell.2020.603421

**Published:** 2020-12-03

**Authors:** Seung-Hyun Ro, Julianne Fay, Cesar I. Cyuzuzo, Yura Jang, Naeun Lee, Hyun-Seob Song, Edward N. Harris

**Affiliations:** ^1^Department of Biochemistry, University of Nebraska-Lincoln, Lincoln, NE, United States; ^2^Department of Neurology, Institute for Cell Engineering, Johns Hopkins University School of Medicine, Baltimore, MD, United States; ^3^Department of Biological Systems Engineering, University of Nebraska-Lincoln, Lincoln, NE, United States; ^4^Department of Food Science and Technology, Nebraska Food for Health Center, University of Nebraska-Lincoln, Lincoln, NE, United States

**Keywords:** Sestrins, environmental stress, aging, metabolic disease, obesity/inflammation, mTORC, ROS, cancer

## Abstract

Proper timely management of various external and internal stresses is critical for metabolic and redox homeostasis in mammals. In particular, dysregulation of mechanistic target of rapamycin complex (mTORC) triggered from metabolic stress and accumulation of reactive oxygen species (ROS) generated from environmental and genotoxic stress are well-known culprits leading to chronic metabolic disease conditions in humans. Sestrins are one of the metabolic and environmental stress-responsive groups of proteins, which solely have the ability to regulate both mTORC activity and ROS levels in cells, tissues and organs. While Sestrins are originally reported as one of several p53 target genes, recent studies have further delineated the roles of this group of stress-sensing proteins in the regulation of insulin sensitivity, glucose and fat metabolism, and redox-function in metabolic disease and aging. In this review, we discuss recent studies that investigated and manipulated Sestrins-mediated stress signaling pathways in metabolic and environmental health. Sestrins as an emerging dynamic group of stress-sensor proteins are drawing a spotlight as a preventive or therapeutic mechanism in both metabolic stress-associated pathologies and aging processes at the same time.

## Introduction of SESTRINs

**FIGURE 1:**
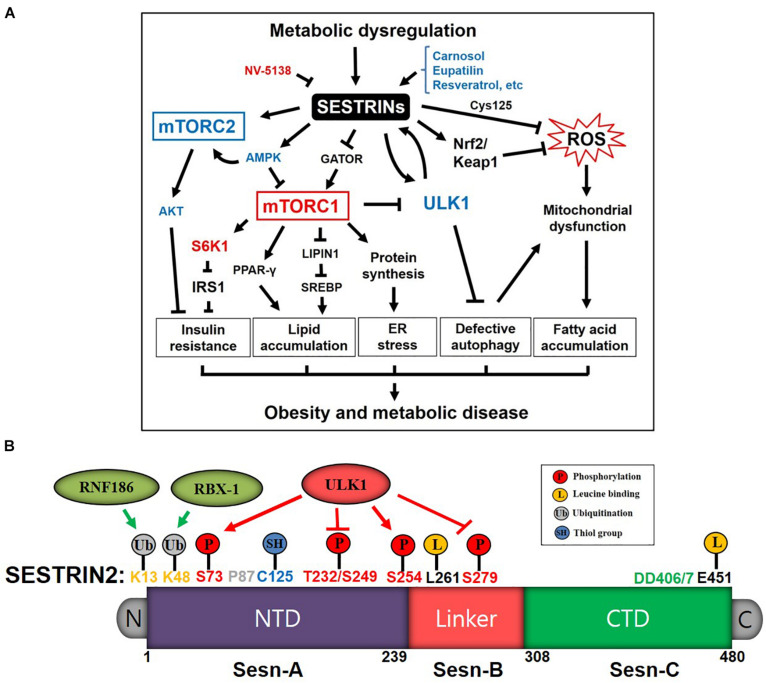
Diagrams depicting the Sestrins-mediated stress signaling pathways and functional domains of Sestrin2 in the regulation of metabolism and disease. **(A)** Schematic diagram of Sestrins/mTORC/ROS pathways in obesity and metabolic diseases induced by metabolic dysregulation stress. Sestrins improve insulin sensitivity by blocking mTORC1/ribosomal protein S6 kinase 1(S6K1) and activating AMPK and mTORC1/AKT pathway. Sestrins also inhibit lipid accumulation and ER stress via mTORC1 inhibition through activating AMPK and binding GATOR2. Sestrins activate autophagy and mitophagy through mTORC1 inhibition and ULK1 activation, which contributes to reducing ROS and removing dysfunctional mitochondria. ULK1 phosphorylates Sestrin2 to induce mitophagy, and phosphorylated Sestrin2 activates ULK1 to further induce autophagy as positive feedback mechanism. Sestrins protect cells, tissues and organs by alleviating stresses and pathologies associated with obesity and metabolic dysfunctions. **(B)** Sestrin2 functional domains (Sesn-A, Sesn-B, and Sesn-C domains) and residues for: (i) protein stability and turnover through ubiquitinations by RNF186 and RBX-1; (ii) redox homeostasis through ROS regulation (Cys125); and (iii) nutrient sensing and regulation through leucine binding, ULK1 activation, and autophagy induction.

SESTRINs (Sestrin1, 2, and 3, gene name: *Sesn*) are a classical family of stress-inducible proteins that regulate metabolism through sensing nutrient level and redox status in cells, tissues and organs. *Sesn1* (aka *PA26*) was originally identified as one of the p53 tumor suppressor target genes in a tetracycline-dependent manner (Buckbinder et al., 1994) and located in chromosome 6q21 (Velasco-Miguel et al., 1999). *Sesn1* is also largely expressed ubiquitously in almost all tissues including the pancreas, kidney, skeletal muscle, lung, and brain (Budanov et al., 2002) and activated in a p53-dependent manner under oxidative and irradiation stresses (Sablina et al., 2005; Budanov and Karin, 2008). *Sesn2* (aka *Hi95*) was identified by microarray in Hif1-independent hypoxia condition of glioblastoma A172 cells and located in chromosome 1p35.3 (Budanov et al., 2002). *Sesn2* is activated by DNA damaging oxidative stress and overnutrition stress in the lung, liver, adipose, kidney and pancreas (Budanov et al., 2002; Lee et al., 2012). *Sesn3* was originally identified by *in silico* data base search and located in chromosome 11q21 (Budanov et al., 2002; Peeters et al., 2003). *Sesn3* is activated by FoxO1 and 3 (Nogueira et al., 2008; Chen et al., 2010; Hagenbuchner et al., 2012) and overexpressed in skeletal muscle, intestine, liver, adipose, kidney, colon and brain (Peeters et al., 2003). However, *Sesn3* is somewhat redundant to *Sesn2* in metabolic function and autophagy induction in liver and colon tissues (Lee et al., 2012; Ro et al., 2016). Although ectopically expressed Sestrin2 protein is mostly found in cytoplasm (Parmigiani et al., 2014), recent studies suggest that Sestrin2 is associated with mitochondria (Kim H. et al., 2020; Kovaleva et al., 2020), nucleus (Tu et al., 2018; Wang L.X. et al., 2020), and endoplasmic reticulum (ER) (Wang L.X. et al., 2020) depending on intra- or extra- cellular stress conditions. Those three Sestrin paralogues have a common antioxidant function that suppresses reactive oxygen species (ROS) (Lee et al., 2013; Wang et al., 2017). Another shared function is that Sestrins activate AMP-activated protein kinase (AMPK), inhibiting the mechanistic target of rapamycin complex 1 (mTORC1) (Parmigiani and Budanov, 2016). Upregulation of mTORC1 has been shown to lead to the accelerated development of several obesity-induced and age-related pathologies, such as lipid accumulation, inflammation, glucose tolerance, insulin resistance, ER stress, mitochondrial dysfunction, protein aggregate formation, cardiac arrhythmia and muscle degeneration (Um et al., 2006; Zoncu et al., 2011). Sestrins are considered to improve obesity-induced and age-related pathologies by inhibiting mTORC1. Protein kinase B (AKT) activation through mTORC2 activation by Sestrins leads to improved insulin sensitivity in obesity and diabetes (Dong, 2015; Kazyken et al., 2019; Knudsen et al., 2020). These pathologies were remedied by inhibition of mTORC1, activation of mTORC2/AKT and the use of antioxidants, which shows that the mTORC- and ROS-controlling functions of Sestrins are indeed important for metabolism and cellular homeostasis (Figure 1A).

By sensing nutrient and redox-activity levels, Sestrins can integrate cellular signal transduction through their conserved residues and translational and transcriptional modifications (Figure 1B). Recent studies of the X-ray crystal structure of Sestrin2 show that it belongs to the family of globin-like α-helix-fold proteins, consisting of 23 helices and no β-sheets, structured by well-conserved Sesn-A [AA 66–239, *N*-terminal structuring domain (NTD)], Sesn-B [AA 254–294, loop linker domain] and Sesn-C [AA308-480, *C*-terminal domain (CTD)] domains among species. Sesn-A and Sesn-C structure are very similar but they have distinctive functions. Sesn-A and Sesn-C are connected by flexible loop linker domain Sesn-B whose functions are not yet known (Kim H. et al., 2015; Ho et al., 2016; Saxton et al., 2016b). Sesn-A has an evolutionarily conserved Cys125 residue with a thiol side chain that has antioxidant function through reducing ROS accumulation (Budanov et al., 2004; Ro et al., 2014a; Kim H. et al., 2015). Knowing the unique structural features of human Sestrin2, several genetic variations and protein modification studies reported the dynamic roles of *Sesn2* in nutrient-sensing, redox-active function, and signal transduction in relation to disease status. K13 and K48 of Sesn-A are ubiquitinated by E3 ligase ring finger protein 186 (RNF186) and ring-box protein 1 (RBX-1) respectively, causing Sestrin2’s degradation in the proteasome (Kumar and Shaha, 2018a; Lear et al., 2019). Using a high-throughput whole-genome single-cell sequencing, Hou et al. (2012) reported that P87S missense mutation of *Sesn2* was found in patients with a certain type of blood cancer, myeloproliferative neoplasms (MPNs), causing aggressive progression of neoplasms. Interestingly, several phosphorylation sites are identified and regulated by unc-51 like autophagy activating kinase 1 (ULK1). ULK1 phosphorylates T232/S249/S279 residues of Sestrin2 which inhibits Sestrin2’s binding with leucine in high nutrient condition (Kimball et al., 2016). Exercise increases the same phosphorylation profile of Sestrin2 and improves skeletal muscle metabolism (Zeng et al., 2017, 2018b). ULK1 also phosphorylates S73 and S254 to activate the autophagic clearance of mitochondria in response to copper-induced oxidative stress (Kim H. et al., 2020). L261 and E451 are identified as essential residues for leucine binding in HEK293 cells (Lee et al., 2016; Saxton et al., 2016a,b; Wolfson et al., 2016). DD406/7 are identified as key residues for GTPase-activating protein (GAP) activity toward recombination activating genes (RAGs) (GATOR) 2 binding so that Sestrin2 can inhibit mTORC1 association with lysosome for its activation (Kim H. et al., 2015; Ho et al., 2016). LSD1, upon deacetylation by histone deacetylase 1 (HDAC1), reduced the methylations of H3K4me2 in the promoter region of *Sesn2*, thereby suppressed the gene expression of *Sesn2* in chronic renal failure (CRF) (Zhou et al., 2020). Jumonji domain-containing protein D3 (JMJD3), a histone demethylase, inhibited the transcription of *Sesn2* by reducing the methylations of H3K27me3 in the promoter region of *Sesn2* in cardiomyopathy (Wang P. et al., 2020). Although dense methylation of CpG islands of *Sesn3* was identified in 20% of endometrial cancers (Zighelboim et al., 2007), the transcriptional regulation (e.g., methylation and demethylation) of *Sesn* genes needs further investigation (Ambrosio et al., 2019). Knowing that Sestrin2 stability, redox-active function, nutrient sensing, and interaction with other proteins can be regulated through those identified residues (Figure 1B), the development of methodology measuring the activity of specific Sestrin2 functional residues would be a promising venue combating against stress-induced pathologies.

Metabolic dysregulation induces Sestrins as a defense mechanism against obesity and metabolic diseases (Lee et al., 2012; Parmigiani and Budanov, 2016; Dalina et al., 2018). Important key features of cellular function are the regulation of insulin sensitivity, lipid/fatty acid (FA) metabolism, ER stress, and autophagy (Lee et al., 2012, 2020; Park et al., 2014). Glucose starvation, ER stress, amino acid deprivation and mitochondrial dysfunction induces Sestrins as a protective mechanism (Park et al., 2014; Ro et al., 2015; Ye et al., 2015; Ding et al., 2016; Garaeva et al., 2016; Saveljeva et al., 2016). Sestrins are central nutrient status sensors and regulates AMPK, mTORC and subsequent downstream metabolic pathways. Sestrin2 improves insulin sensitivity, prevents excessive lipid accumulation and alleviates ER stress by inhibiting mTORC1 through AMPK activation (Budanov and Karin, 2008) and GATOR binding (Chantranupong et al., 2014; Parmigiani et al., 2014; Kim J.S. et al., 2015) (Figure 1A). Tao et al. (2015) reported that Sestrin3 prevents insulin resistance by activating mTORC2/AKT pathways in the liver. Sestrin2 also activates AKT through mTORC2 and GATOR2 (MIOS/WDR24/SEH1L/WDR59/SEC13) bindings (Kowalsky et al., 2020). Sestrins can eliminate ROS by inducing antioxidants enzymes by activating nuclear factor erythroid 2-related factor 2 (Nrf2) through p62 (gene name: SQSTM1)-dependent degradation of Kelch-like ECH-associated protein 1 (Keap1) pathways, which can reduce the damaged mitochondria, improve fat metabolism, and maintain mitochondrial function in mammalian cells (Bae et al., 2013). Knockdown of Sestrin2 aggravates the atherosclerotic process in human umbilical vein endothelial and THP-1 cell lines and C57BL/6 mice by increasing pro-inflammatory response (Hwang et al., 2017). Sestrin2 suppresses sepsis by inhibiting prolonged nod-like receptor (NLR) family pyrin domain containing 3 (NLRP3)-inflammasome activation (Kim et al., 2016) and protects macrophage by inhibiting TLR-induced pro-inflammatory signaling (Yang et al., 2015). Lysine-specific demethylase 1 (LSD1) suppresses oxidized low-density lipoprotein (Ox-LDL)-induced inflammation by promoting Sestrin2-mediated PI3K/AKT/mTOR pathway activation (Zhuo et al., 2019). Recently, Navitor Pharmaceuticals developed Sestrin2-specific inhibitor, a leucine analog NV-5138, and try to treat the major depressive disorder (MDD) by activating mTORC1 in the brain (Hasegawa et al., 2019; Kato et al., 2019; Sengupta et al., 2019). However, one of the main causes of obesity, inflammation and metabolic disease is the hyperactivation of mTORC1, so the development of Sestrin2-specific activator compound or drug would be a potential therapeutic (Dong, 2015). Indeed, several recent studies suggest that synthetic chemicals or naturally occurring compounds induces Sestrins gene expression as adaptive defense mechanisms upon various cellular damaging stresses (Sanchez-Alvarez et al., 2019). In the examples of *Sesn2* induction, synthetic drugs such as eupatilin (Jegal et al., 2016), topotecan (TPT) (Chen et al., 2015), tanshinone IIA (TIIA) (Yen et al., 2018), and cheliensisin A (ChlA)-F (Hua et al., 2018) induce autophagy by increasing *Sesn2* expression as cellular defense upon oxidative stress and DNA damage. The naturally occurring resveratrol inhibits hepatic lipogenesis through *Sesn2* induction (Jin et al., 2013), thus keeping fatty liver in check. Nelfinavir/bortezomib (Bruning et al., 2013) and protopanaxadiol (PPD) (Jin et al., 2019) are known to induce *Sesn2* through protein kinase R (PKR)-like endoplasmic reticulum kinase (PERK)/ cyclic AMP-dependent transcriptional factor (ATF) 4 activation upon ER stress. Albeit the increasing therapeutic potential of allosteric modulation of Sestrin2 is promising, the unexpected side effects should be thoroughly examined by animal studies and human clinical trials.

## SESTRINs in Nutrient Stress-Sensing and Metabolic Dysfunction

In modern westernized society, excessive consumption of fat and sugar combined with sedentary life style causes an exponential increase in the number of patients with obesity and metabolic diseases such as diabetes, cardiovascular disease, and cancer (Bidwell, 2017; Pallazola et al., 2019; Leisegang et al., 2020). Hyperactivation of mTORC1 is one of the underlying mechanisms causing insulin resistance, lipid accumulation, ER stress, defective autophagy function and mitochondrial dysfunction (Um et al., 2006; Zoncu et al., 2011; Tao and Xu, 2020). Here, we reviewed Sestrins, potent inhibitors of mTORC1, and its mechanism for preventing nutrient-associated stress and metabolic dysfunctions.

After *Sesn1* was originally identified as one of the p53 target genes in the late 90’s (Velasco-Miguel et al., 1999), *Sesn2* was also identified under the control of p53 to induce autophagy under DNA damage and oxidative stress (Maiuri et al., 2009). When S18 of p53, a phosphorylated target of ataxia-telangiectasia mutated (ATM) kinase, was mutated to Ala in p53 ^*S18A*^ knock-in mice, these mice developed a diabetic phenotype with abnormal glucose homeostasis and insulin resistance within 6 months of age. This phenotype was alleviated with ectopically overexpressed Sestrin2, suggesting that Sestrin2 has an anti-diabetic effect (Armata et al., 2010). Indeed, Sestrin2 and 3 have a protective effect against obesity-induced metabolic dysfunction and insulin resistance, which is revealed by investigating the liver and adipose tissues of *Sesn2/3* double knockout (DKO) mice (Lee et al., 2012). *Sesn2* ablation exacerbates obesity-induced mTORC1-S6K activation, and moreover, the concomitant ablation of *Sesn2* and *Sesn3* provokes hepatic mTORC1-S6K activation and insulin resistance even in the absence of nutritional overload and obesity (Lee et al., 2012). Although *Sesn2* is suggested to be dominant over *Sesn3* in the previous adipose and liver metabolism studies, each Sestrin have unique stress-specific regulatory functions. Sestrin2-AMPK axis alleviates hyperglycemia-induced glomerular injury caused by high glucose, high level of ROS and decreased level of nitric oxide (Eid et al., 2013). Sestrin3 reduces lipid accumulation of chronic alcohol-induced liver injury (Kang et al., 2014). And this demonstrates an important homeostatic function for the stress-inducible Sestrin protein family in the control of mammalian lipid and glucose metabolism. It is shown that *Sesn3* liver-specific knockout (KO) mice exhibit insulin resistance and glucose intolerance, and *Sesn3* transgenic mice were protected against insulin resistance induced by a high fat diet (HFD). Also, it has been demonstrated that the *Sesn3* insulin-sensitizing effect is largely independent of AMPK, and *Sesn3* can activate AKT via mTORC2 to regulate hepatic insulin sensitivity and glucose metabolism (Tao et al., 2014). However, the study using adipose-specific Sestrin2 overexpressing mice (PG*-Sesn2*) suggests that Sestrin2 interferes with brown adipose tissue (BAT)-specific expression of uncoupling protein 1 (UCP1) through suppression of ROS-mediated p38 MAPK activation, and resulted in the undesirable accumulation of lipids in BAT (Ro et al., 2014a). These results indicate that maintaining the physiological level and activity of Sestrins in each tissues are critical for metabolic homeostasis and may provide a new therapeutic approach for the prevention of obesity, insulin resistance and diabetes.

Regular physical exercises benefit human health by protecting humans against obesity and metabolic dysfunctions including insulin resistance, glucose intolerance and galactose malabsorption. However, the mechanism of how Sestrins are induced by exercise and their beneficial effects on human health are not completely understood (Nascimento et al., 2013). AMPK and TORC1 are cellular energy sensors that respond in a reciprocal manner to environmental conditions such as nutrient supply or cellular stress to control metabolism and growth in insulin-sensitive tissues like skeletal muscle. A recent study suggests that exercise induces direct interaction between Sestrin2 and AMPK and improves insulin sensitivity through autophagy induction (Li et al., 2017). The expression levels of both Sestrin2 and 3 along with autophagy activity were increased in skeletal muscle of HFD-fed mice after long-term physical exercises (Liu et al., 2015). Both Sestrin2 and 3 directly bind with AMPK and promotes glucose uptake in skeletal muscle in wild type (C57BL/6) and but not in *AMPKα2^–/–^* mice (Morrison et al., 2015; Wang et al., 2018). Sestrins were directly induced by exercise and its phosphorylation by ULK1 kinase was observed along with autophagy induction which improved metabolic parameters, prevented atrophy and maintained mitochondrial mass in muscle (Li et al., 2019; Xu et al., 2019; Kim M. et al., 2020; Segales et al., 2020). Acute exercise increased Sestrin1 whereas chronic exercise up to 4 weeks increased both Sestrin1 and 2 in mice suggesting that Sestrins have distinct functions on the intensity and duration of the exercise (Crisol et al., 2018). In these studies, Sestrins were suggested as plausible cellular mechanism by which exercise protects against metabolic diseases.

Chronic hyperactivation of mTORC1 is one of the hallmarks of tumor proliferation and growth as cancer cells rapidly synthesize proteins, lipids and cellular components in response to metabolic dysregulation, hypoxic environment and genetic mutation stresses (Guertin and Sabatini, 2007; Jones and Thompson, 2009). mTORC1-specific inhibitors such as rapamycin, metformin and aspirin suppresses intestinal and breast cancer initiation and growth (Faller et al., 2015; Pulito et al., 2017). mTORC1/2 inhibitor torin suppresses hepatocellular carcinoma (HCC) cell growth (Wang et al., 2015). Although mTOR inhibitors were widely used previously, efficacy and safety on cancer patients are controversial due to their side effects (Kalender et al., 2010; Blagosklonny, 2011; Din et al., 2012). Knowing that Sestrins are specific mTORC1 inhibitors through AMPK activation and GATOR binding, Sestrin-based therapeutics are emerging as a novel anti-cancer and tumor treatment with less off-target effects. Here, we have reviewed how Sestrins act on tumor suppression and progression in two different aspects. Recent studies identified that Sestrin2 is down-regulated in colorectal cancer and overexpression of Sestrin2 inhibited colon carcinogenesis through mTORC1 inhibition (Wei J.L. et al., 2015; Wei et al., 2017; Ro et al., 2016). Loss of heterozygosity in *Sesn1* (6q21) and *Sesn2* (1p35) is frequently detected in human solid tumors (Ragnarsson et al., 1999; Velasco-Miguel et al., 1999). Sestrins are targets of p53 which, under normal conditions, prevents the outgrowth of cancer cells. There is evidence in the literature which shows that chronic stress-induced inactivation of the p53 target, Sestrin family, promotes the outgrowth of cancer cells. For some examples, inactivation of p53 and repression of *Sesn1/3* contribute to Ras oncogene-induced ROS accumulation and genetic instability in immortalized embryonic fibroblasts (Kopnin et al., 2007). *Sesn2*-deficient mouse embryonic fibroblast (MEF) cells were significantly more susceptible to oncogenic stresses (Budanov and Karin, 2008). *Sesn2* inhibition promotes A549, human a non-small cell lung carcinoma, cell growth (Sablina et al., 2005), and its expression level is negatively correlated with the lung cancer survival rate (Chae et al., 2020). *Sesn3* induces apoptosis of human non-small cell lung cancer (NSCLC) cells in dietary compound cucurbitacin B treatment (Khan et al., 2017). *Sesn3*-deficiency developed severe HCC via hedgehog pathway activation in *Sesn3* KO mice (Liu et al., 2019). In the recent study, knockdown of *Sesn2* promoted the cell growth, migration, and oxidative stress of HEC-1A and ishikawa endometrial cancer cells through mTOC1 hyperactivation (Shin et al., 2020). These findings suggested that Sestrins have a tumor suppressive function through downstream activation by p53.

On the contrary to Sestrins’ tumor suppressive role, other studies suggest that Sestrins would promote cancer cell proliferation and growth in nutrient-deficient or oxygen- limited (hypoxic) conditions because Sestrins can activate autophagy and reduces ROS in the hypoxic cancer microenvironment (Maiuri et al., 2009). *Sesn2* promotes tumorigenesis and chemo-resistance under Ultraviolet B (UVB) stress and chemo-therapeutics by activating AKT in human squamous cell carcinomas (SCCs) and melanoma cells (Zhao et al., 2014). Both UVB and UVA induce *Sesn2* upregulation in melanocytes and melanoma cells suggesting the oncogenic role of *Sesn2* in melanoma skin cancer (Zhao et al., 2017). The gene expression of *Sesn2* upregulated by miR182-5p suppression in arsenic treatment functions as an antioxidant in Uppsala 87 malignant glioma (U87MG), human lung adenocarcinoma H1299, and A549 cell lines (Lin et al., 2018). *Sesn2* would promote colorectal cancer cell growth in the iron-rich environment by suppressing ROS (Kim et al., 2019). Sestrins promote anchorage-independent (anoikis resistance) growth of human endometrial cancer cells (Kozak et al., 2019). Despite Sestrins’ contribution on early tumor growth, Sestrins suppress late stages of carcinogenesis in a mouse lung cancer model and A549 cells (Ding et al., 2019). Carnosol, a dietary diterpene from rosemary, induces Sestrins as antioxidants in apoptotic death of HepG2 human hepatoma and HCT116 and SW480 human colorectal carcinoma cells (Tong et al., 2017; Yan et al., 2018). Sestrins promote cell death of breast cancer cells upon irradiation and DNA-damaging drug treatment through mTORC1 inhibition (Sanli et al., 2012). On the other hand, Sestrins may block oxidative damage-associated chemotherapy by reducing ROS (Budanov and Karin, 2008; Hagenbuchner et al., 2012). Sestrins were upregulated upon drug treatments in various type of pre- and mature-tumors as cell survival mechanism: head and neck cancers (Won et al., 2019), non-alcoholic steatohepatitis (Huang et al., 2020), HCC (Dai et al., 2018), osteosarcoma (Yen et al., 2018), acute pancreatitis (Norberg et al., 2018), colitis (Ro et al., 2016), bladder cancer (Hua et al., 2018), and prostate cancer (Fu et al., 2018; Shan et al., 2019). The distinctive roles of Sestrins as tumor suppressor or oncogene in the early or late stages of cancers need further investigation (Sanchez-Alvarez et al., 2019). These results might lead to a novel anti-tumor therapy through the cancer type- and cancer stage-dependent administration or induction of Sestrins.

## SESTRINs in Environmental Stress and Aging

Aging or shortening of lifespan is closely connected with the exposure to oxidative stress, DNA damaging agents, losing immunity, and metabolic dysregulation (Lee et al., 2013; Wei Y. et al., 2015; Srivastava, 2017; Dalina et al., 2018; Fan et al., 2020; Sun et al., 2020). As human life expectancy has extended, the importance of healthy aging or elderly health is increasing and drawing significant attention among the general public. Since Sestrins are known to have anti-aging effect by removing oxidative stress, improving cellular metabolism and immunity, their therapeutic role in healthy aging and elderly health has been emerging subject of scientific studies (Budanov et al., 2010; Lee et al., 2010a; Budanov, 2011; Martyn and Kaneki, 2020). In this section, we have reviewed the essential function of Sestrins as a barometer of stress and indicator of human health at the molecular level focusing on the recent reports.

**FIGURE 2:**
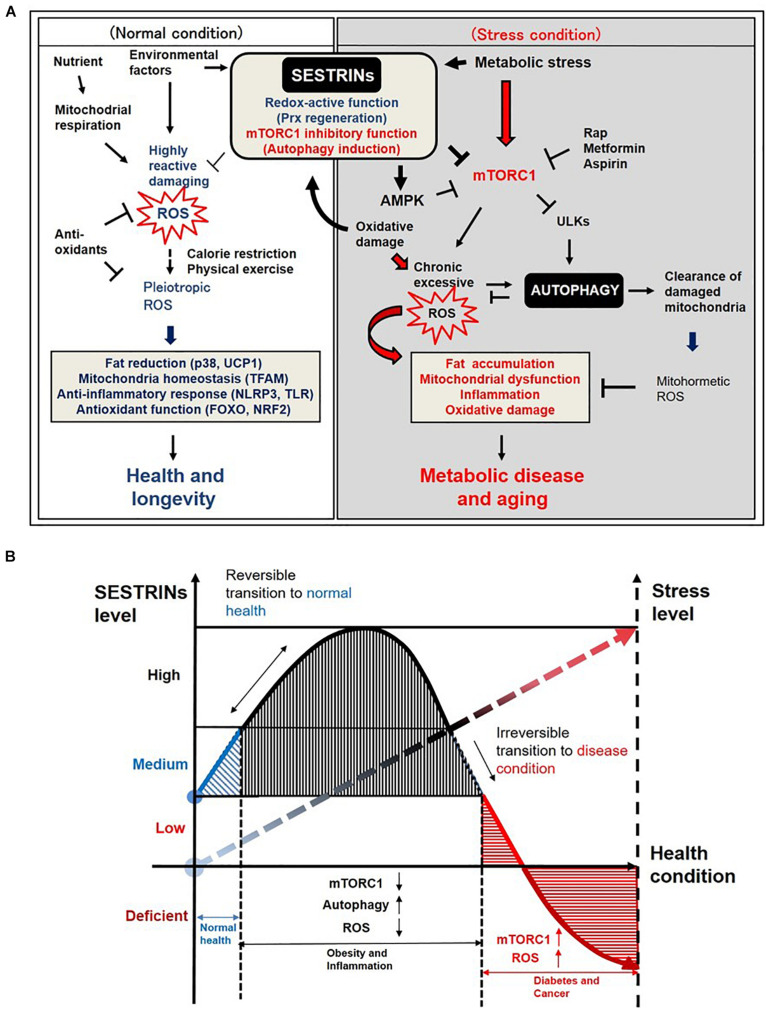
Diagrams depicting the roles of Sestrins in managing stress levels and health conditions. **(A)** Diagram depicting the possible roles of Sestrins in the regulation of ROS and autophagy in mammalian cells. (*Normal condition*) Redox-active function of Sestrins may suppress highly reactive damaging ROS and maintain pleiotropic ROS level such as mitohormesis in mammalian cells under normal nutrient and environmental factors. (*Stress condition*) Sestrins are induced by metabolic or genotoxic stresses such as overnutrition and oxidative damage. Chronic activation of mTORC1 may induce chronic excessive ROS, which results in aging-associated phenotypes such as fat accumulation, mitochondrial dysfunction, inflammation, and oxidative damage in mammalian cells. However, Sestrins inhibit mTORC1 activity and subsequently induce or regulate autophagy activity in the cells and tissues. Activated autophagy removes oxidized proteins and also clear damaged mitochondria which are producing excessive harmful ROS. Overall, Sestrins-mediated autophagy regulates the ROS level to trigger redox adaptations under stress conditions. The underlying mechanism of how pleiotropic level of ROS is maintained by Sestrins-mediated autophagy signaling pathway needs further investigation. **(B)** Graphic diagram explaining the correlation between Sestrins and stress levels in health and disease conditions.

Sestrins possess two important functions that contribute to protecting cells, tissues and organs against environmental stress and aging (Figure 2A). First, Sestrins function as antioxidants, promoting regeneration of peroxiredoxins (Prxs), one of the major ROS scavengers in cells (Budanov et al., 2004; Ro et al., 2014a, 2015). Initially, mammalian Sestrins shows very low similarity to any proteins performed by the basic local alignment search tool (BLAST) search and position-specific scoring matrix (PSSM) analysis (Budanov et al., 2004, 2010). However, residues 100–175 AA shared sequence and structure homology with alkyl hydroperoxidase D (AhpD) which is a *Mycobacterium tuberculosis* hydrogen peroxide reductase and regenerating oxidized bacterial Prxs AhpC by ROS or reactive nitrogen species (RNS). AhpD is a disulfide reductase but Sestrin2 might be a cysteine sulfinyl reductase regenerating oxidized Prxs (Budanov et al., 2004) although it remains controversial (Woo et al., 2009). Further investigation on the role of Sestrins in anti-oxidant defense related to Prxs which confer thiol oxidation of a majority of cytosolic proteins including aurora kinase A (Perkins et al., 2015; Stocker et al., 2018; Byrne et al., 2020) and promote longevity by regulating nutrient signaling protein kinase A (Bodvard et al., 2017; Roger et al., 2020) is warranted to resolve this controversy. Sestrin2 reduces alkylhydroperoxide radicals and ROS accumulation through Cys125 (Ro et al., 2014a; Kim H. et al., 2015). Second, Sestrins stimulate autophagy by inhibiting rapamycin-sensitive mTORC1 comprising of mTOR and Raptor (Kim et al., 2002), through activating AMPK or through binding with GATOR2 (Kim H. et al., 2015; Kim J.S. et al., 2015). Inducing autophagy also contributes to the suppression of ROS, because it eliminates dysfunctional mitochondria that produce the pathogenic level of ROS (Salminen et al., 2012; Ashrafi and Schwarz, 2013). Sestrins also induce the gene expression of antioxidant enzymes via Nrf2 activation through autophagy-mediated degradation of Keap1 (Bae et al., 2013). Sestrin2 associates with autophagy kinase ULK1 and binds with autophagy adaptor protein p62 to activate autophagy (Ro et al., 2014b; Lim et al., 2015). This autophagy activity was significantly blocked when *Sesn2* KO MEF cells were treated with rapamycin as an autophagy inducer (Lee et al., 2012). Recent studies indicate that Sestrin2 activates the autophagic clearance of damaged mitochondria through Parkin-ULK1-Beclin1 activation (Kumar and Shaha, 2018b) and also induces mitophagy in macrophages and renal tubular cells (Ishihara et al., 2013; Kim et al., 2016). ULK1 phosphorylates Sestrin2 at S73 and S254 to induce mitophagy upon copper-catalyzed oxidative stress (Kim H. et al., 2020). Although recent study suggests that Sestrin2 is colocalized with mitochondria and regulates some mitochondrial functions (Kovaleva et al., 2020), its direct translocation into mitochondria and involvement in maintaining mitochondrial antioxidant enzymes function and ROS level remain unknown.

Given the existence of three distinct paralogues in mammalian Sestrins (Sestrin1/2/3), *Drosophila melanogaster (D. melanogaster)* and *Caenorhabditis elegans (C. elegans)* have single Sestrin in their genomes, namely *dSesn* and *cSesn*, respectively. Like mammalian Sestrins, *dSesn* is highly expressed in cardiac and skeletal muscle tissues and fat body (Lee et al., 2010b). *dSesn*-null mutant *D. melanogaster* significantly exhibited multiple age-associated degenerative pathologies, including mitochondrial dysfunction, lipid accumulation, cardiac dysfunction, and muscle degeneration. Among these pathologies, mitochondria dysfunction and accompanying fat accumulation was well pronounced (Lee et al., 2010b), which is well correlated with the muscle- and fat body-specific expression patterns of *dSesn*. These pathologies were ameliorated by administering antioxidants and/or autophagy inducers such as metformin and rapamycin (Lee et al., 2010b). Other genetic results also suggest that the ROS- and autophagy-regulating functions of Sestrins are important for muscle and fat body homeostasis in *D. melanogaster* (Lee et al., 2010a, b). Considering that Sestrins are potent inducers of autophagy (Maiuri et al., 2009) and that *dSesn*-deficient flies and *Sesn2/3*-deficient mice are defective in autophagy function (Lee et al., 2010b, 2012; Bae et al., 2013), it is highly speculated that Sestrins-regulated autophagy is beneficial for metabolic tissue homeostasis and protective against metabolic dysfunctions. *dSesn* reduces oxidative stress from chromium [Cr(IV)]-induced neuronal cell death (Singh and Chowdhuri, 2018). Recent studies also reported that *C. elegans*, lack sulfiredoxin (Srx) but encode *cSesn* (*Sesn1* orthologue), remove oxidative stress and increase longevity (Thamsen et al., 2011; Yang et al., 2013). Since both ROS accumulation and autophagy inhibition are among the common molecular pathologies underlying human metabolic dysfunctions and aging, Sestrins may also have tissue-specific protective roles distinctively from conserved functions in most metabolic tissues (e.g., adipose, brain, colon, liver, cardiac muscle, and skeletal muscle) which are very sensitive to redox regulation and aging (Liu et al., 2015; Li et al., 2017).

Recent studies suggest that Sestrins improve longevity and elderly health by suppressing ROS, regulating autophagy activity and protecting metabolic dysfunction in muscle, heart and brain (Rai et al., 2018; Chen et al., 2019; Cordani et al., 2019; Fan et al., 2020; Liu et al., 2020; Segales et al., 2020; Sun et al., 2020). The level of Sestrins expression is downregulated in aged men although it has no clear correlation with autophagy activity, mTORC1 activity and antioxidant regulation (Zeng et al., 2018a). Both *Sesn1* and *2* levels are significantly decreased in patients with sarcopenic muscle disease (Rajan et al., 2020). Resistant exercise but not dietary protein increased the *Sesn2* level and its phosphorylation in the skeletal muscle of elderly humans (Zeng et al., 2018b) and aged mice (Lenhare et al., 2017). The following are examples in the way Sestrins are involved with symptoms of aging. *Sesn2* is induced in patients with hypertension (Fang et al., 2020) and overexpressed *Sesn2* was protective against cardiomyopathy (Li et al., 2019; Wang P. et al., 2019) and cardiac dysfunction via extracellular signal-regulated protein kinases (ERK)1/2 inhibition (Dong B. et al., 2017) and liver kinase B (LKB)1-mediated AMPK activation (Morrison et al., 2015). *Sesn2* also prevents age-related intolerance to myocardial infarction via AMPK/peroxisome proliferator-activated receptor gamma coactivator 1-alpha (PGC-1α) pathway (Quan et al., 2018), ischemia and reperfusion injury (Quan et al., 2017), atrial fibrillation (Dong Z. et al., 2017), radiation-induced myocardium damage (Zeng et al., 2016) in human and rodent models. These studies indicate that maintaining the homeostatic level of *Sesn2* is protective against cardiovascular aging (Liu et al., 2020; Sun et al., 2020). *Sesn2* promoted autophagy in the brain and prevented neurodegenerative diseases by alleviating oxidative stress (Chen et al., 2019; Luo et al., 2020). *Sesn2* protects neuronal cells from cerebral ischemia-reperfusion injury by increasing Nrf2, Srx1, and thioredoxin (Trx) 1 (Zhang and Zhang, 2018). *Sesn3* positively regulates a pro-convulsant gene network in the human epileptic hippocampus (Johnson et al., 2015). As a non-canonical function of the Sestrin family, Sestrins improve life-long immunity and extend longevity through enhancing natural killer cell function (Pereira et al., 2020) and vaccine responsiveness (Lanna et al., 2017). *Sesn3* promotes the generation of macrophage-mediated helper T cells in colitis (Ge et al., 2020). Taken together, Sestrins’ canonical and non-canonical functions contribute to the protection against the aging process and extend healthy lifespan.

## Perspective on SESTRINs as Stress Sensing-Proteins in Metabolic Disease and Aging

During the most recent decade, the research on Sestrin2 was increased exponentially according to PubMed and google scholar analysis data (Wang L.X. et al., 2019). Unlike other kinases or transcription factors, a group of Sestrins protein family confers unique dual functions: sensing nutrient stress and reducing oxidative stress. These unique features of stress-inducible protein Sestrins are becoming an intriguing theme among research groups around the world. It is still debatable that maintaining low level of ROS or RNS is actually beneficial to the human body similar to vaccination rather than completely removing all (Kenny et al., 2019; Klaus and Ost, 2020). For example, mitohormesis refers to maintain physiological levels of ROS or RNS which would benefit but could harm tissues and organs at an excessive level (Yun and Finkel, 2014; Barcena et al., 2018; Palmeira et al., 2019). In the current literature, ROS is emerging as a pleiotropic physiological signaling agent in addition to their characteristics as highly reactive damaging species (Holmstrom and Finkel, 2014; Sies and Jones, 2020). For instance, metformin, a well-known anti-diabetic drug, extends the life-span of worms through the production of low levels of mitochondrial ROS and Prxs PRDX-2 (De Haes et al., 2014). Low levels of H_2_O_2_ extends the life-span of yeast in a Prx-dependent manner (Goulev et al., 2017) and an endogenous amino-acid metabolite, namely *N*-acetyl-L-tyrosine (NAT), was recently shown to reduce tumor growth in mice via mitohormesis (Matsumura et al., 2020). Sestrins could be functionally similar to Prx by fine-tuning a pleitropic level of ROS, which might be beneficial for human health, but the underlying mechanism needs further interrogation. Another intriguing speculation is that Sestrins can be induced by various stress conditions in the earlier inflammation and metabolic dysfunction stage, then they act as a cellular defense mechanism by suppressing mTORC1, inducing autophagy and removing ROS. When the stress level goes over the threshold which indicates the metabolic diseases and aging conditions, the Sestrins level goes down in tissues and organs and human can suffer from the irrevocable conditions confronting pain and death (Figure 2B). Initially, the upregulation of Sestrins is observed as a defense mechanism in the tissues under metabolic stress and inflammation or during predisposition stage of metabolic syndrome (Lee et al., 2012; Ro et al., 2016), as chronic stress conditions sustain, the level of Sestrins is dramatically suppressed in the etiology of disease conditions such as type 2 diabetes, dyslipidemia, colon cancer development and muscle aging progression (Wei J.L. et al., 2015; Ro et al., 2016; Kim M. et al., 2020; Segales et al., 2020; Sundararajan et al., 2020). In recent human studies, secreted Sestrin2 level in the serum of obese children, diabetic nephropathy patients, and elderly sarcopenia adults is significantly lower and associated with metabolic dysfunction (Nourbakhsh et al., 2017; Mohany and Al Rugaie, 2020; Rajan et al., 2020), suggesting that the expression or the secretion of Sestrin2 is somehow blocked in the disease state. Measuring tissue- or individual patient-specific Sestrins level could be used as a direct health or stress sensors, and proper timely administration of active Sestrins would be emerging preventive or therapeutic methods for those who are suffering from metabolic diseases and aging-associated pathologies.

## Author Contributions

All authors contributed to the article and approved the submitted version.

## Conflict of Interest

The authors declare that the research was conducted in the absence of any commercial or financial relationships that could be construed as a potential conflict of interest.
